# Supplementary material for: Sex-Related Differences in Left Atrial Low-Voltage Areas According to CHA2DS2-VA Scores among Patients with Atrial Fibrillation
Source: J Clin Med. 2022 May 31;11(11):3111. doi: 10.3390/jcm11113111 (PMC9181075; doi:10.3390/jcm11113111)
Supplement: Supplementary file 1 [file jcm-11-03111-s001.zip › jcm-1733848-supplementary.pdf]

Supplementary Table S1. LVA extension by CHA_2DS_2-VA score.

**CHA_2DS_2-VA score**	0 (n=244)	1 (n=156)	2 (n=97)	3 or 4 (n=52)	5 (n=4)	
**Proportion of LVAs, (%)**	9.1 [4.8–15.9]	9.8 [5.4–15.3]	9.1 [5.8–17.3]	12.0 [5.4–16.4]	19.1 [17.0–36.5]	0.198

LVAs; Low-voltage areas

Supplementary Table S2. Difference between sexes according to low-voltage area proportions by CHA_2DS_2-VA scores.

CHA_2DS_2-VA score	Male sex		Female sex		*p*-value
	Number of patients	Proportion of low-voltage areas (%)	Number of patients		
0	200	9.3 [4.9–15.9]	44	8.1 [4.8–15.8]	0.957
1	126	10.0 [4.7–15.0]	30	9.4 [5.8–17.8]	0.536
2	78	8.6 [5.8–14.8]	19	9.6 [6.5–26.9]	0.398
3 or 4	36	10.1 [4.7–15.1]	16	15.8 [9.2–32.1]	0.027
5	4	19.1 [17.0–36.5]	0		

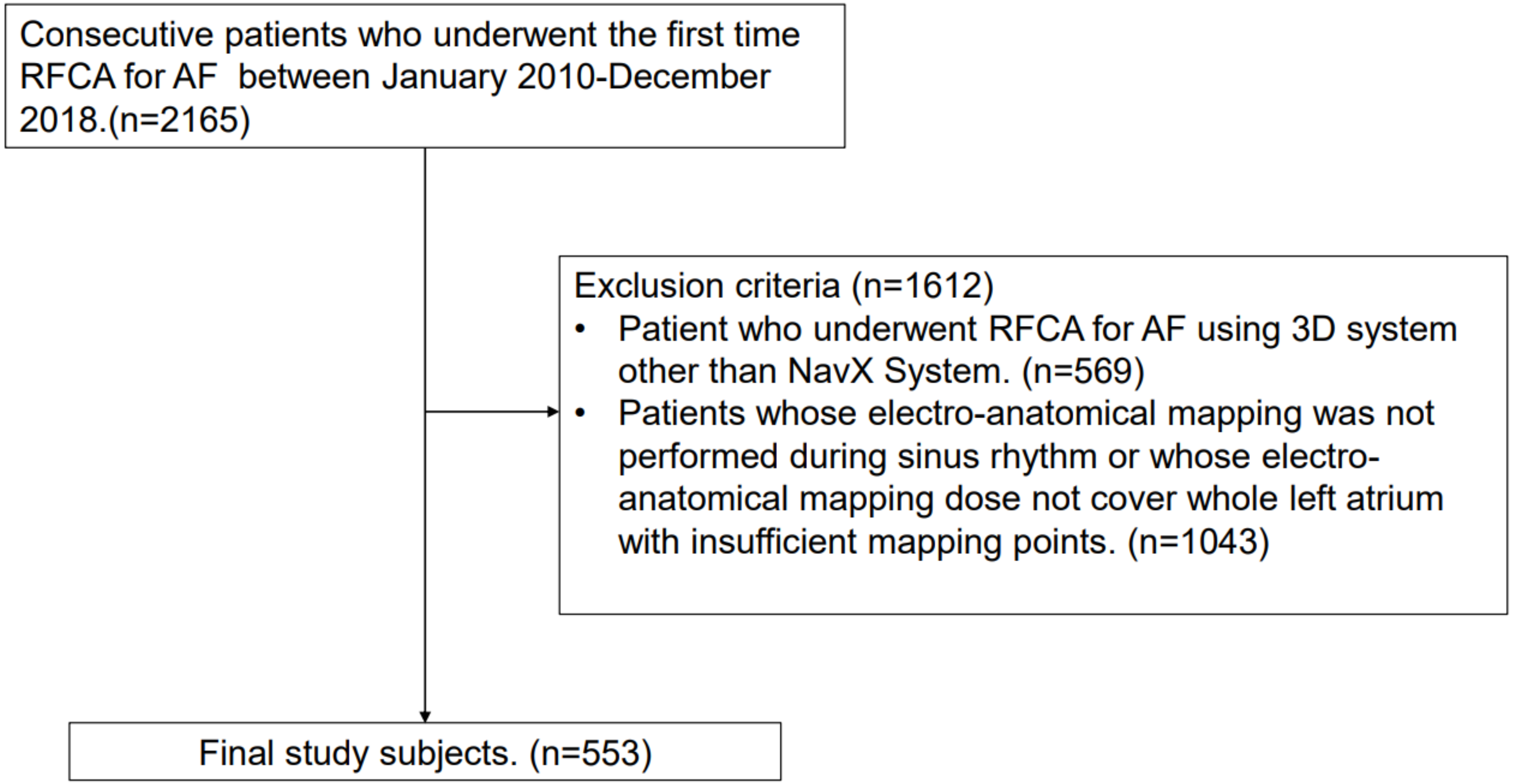

Supplemental Figure S1. Flow chart for study enrollment.
